# Association of thyroid function test abnormalities with preeclampsia: a systematic review and meta-analysis

**DOI:** 10.1186/s12902-022-01154-9

**Published:** 2022-09-26

**Authors:** Mahboubeh Hajifoghaha, Saeed Hosseini Teshnizi, Sedighe Forouhari, Mohammad Hossein Dabbaghmanesh

**Affiliations:** 1 Department of Midwifery, School of Nursing and Midwifery, Shiraz University of Medical Sciences, Shiraz, Iran; 2 Department of Community Medicine, School of Medicine, Hormozgan University of Medical Sciences, Bandar-Abbas, Iran; 3 Infertility Research Center, Shiraz University of Medical Sciences, Shiraz, Iran; 4 Endocrinology and Metabolism Research Center, Shiraz University of Medical Sciences, Shiraz, Iran

**Keywords:** Pregnancy, Preeclampsia, Thyroid, Thyrotropin, Meta-analysis

## Abstract

**Background:**

Preeclampsia is a life-threatening disorder during pregnancy and postpartum periods. Preeclampsia can affect the activity of many organs. It is very important because if this disorder is associated with changes in thyroid function, it can affect the results of maternal and fetal tests. Accordingly, the aim of this meta-analysis study was to assess the abnormalities in thyroid function tests in preeclampsia.

**Methods:**

Studies were selected through a systematic search of the MEDLINE/PubMed, Scopus, Web of Science Core Collection, and Google Scholar databases in 31st August 2021. Also, reference lists of review articles and relevant studies were manual-searched to identify other potentially eligible studies. English studies that compared TSH, T4 and T3 of normal pregnant with preeclamptic women (Known to be normotensive before pregnancy; gestational age 20 week or more; singleton pregnancy; no previous history of thyroid dysfunction) were screened. Data sets were screened for eligibility by two independent reviewers. Articles were assessed by the Newcastle–Ottawa Scale. The Grading of Recommendations Assessment, Development and Evaluation (GRADE) approach was used for quality assessment of evidence on outcome levels.

**Results:**

After reviewing 886 published studies, 63 observational studies were selected and used for this meta-analysis. The study population included 21,528 pregnant women. The findings revealed that TSH (SMD = 1.70, 95%CI: 1.39 to 2.02; *p* < 0.001) was significantly higher in preeclamptic women. TT4 (SMD = -0.82, 95%CI: -1.16, -0.49; p < 0.001), TT3 (SMD = -0.88, 95%CI: -1.36 to -0.41; *p* < 0.001) and FT3 (SMD = -0.59, 95%CI: -0.91 to -0.27; p < 0.001) were less in preeclamptic women. There was no statistically significant difference in FT4 between two groups (SMD = 0.002, 95%CI: -0.27 to 0.27; *p* = .990). The results of publication bias and sensitivity analysis confirmed the reliability and stability of this meta-analysis. The quality of evidence was regarded as moderate, low, and very low for these risk factors according to the GRADE approach.

**Conclusions:**

Findings of this meta-analysis indicated preeclamptic women were more at risk of changes in thyroid function tests. In order to prevent thyroid disorders, it is recommended that thyroid function tests be performed in women with pre-eclampsia.

**Supplementary Information:**

The online version contains supplementary material available at 10.1186/s12902-022-01154-9.

## Background

Preeclampsia complicates 2–8% of pregnancies and is one main cause of maternal and neonatal mortality and morbidity worldwide [1, 2]. The American College of Obstetricians and Gynecologists (ACOG) defined preeclampsia in 2017 as having a blood pressure of more than 140/90 mmHg at intervals of four hours after the 20th week of pregnancy, as well as proteinuria greater than or equal to 300 mg in 24 h of urine collection [3]. Preeclampsia is a multi-systemic disorder [4].

During pregnancy, the physiological changes of the thyroid gland are completely normal and incompatibility with these changes leads to dysfunction of the thyroid gland [5]. Naturally, thyroid hormones increase by 40–100% to meet the needs of both mother and fetus [6]. In a normal pregnancy, Thyroid Stimulating Hormone (TSH) increases due to an increase in concentration of Human Chorionic Gonadotropin (HCG) [7]. Total Thyroxine(TT4) and Total Triiodothyronine(TT3) concentrations increase rapidly, while Free Triiodothyronine and Free Thyroxine (FT3 and FT4) increase at a slower rate [6]. Most serum thyroid hormones are protein bound and only 0.2% of T3 and 0.02% of T4 are free. About 45- 70% of thyroid hormones bind to thyroxine-binding globulin (TBG) and the rest to trans-thyrotin and albumin. [7].

Preeclampsia affects the function of many organs in the body, including the thyroid gland [8]. In general, the findings were not the same in all studies. For example, in one study, despite increased in TSH levels, T3 and T4 levels did not change notably in women with preeclampsia [9]. The findings of two studies revealed that TSH, TT4, and TT3 were not differ significantly between preeclamptic and normal pregnant women [3, 10]. The American Thyroid Association (ATA) guidelines report that there is no association between changes in thyroid function tests and preeclampsia [11]. Some findings resulted that preeclamptic women had higher incidence of the increase in TSH and low T4 in a comparison with normal pregnant women [10].

Some studies have shown that 29.3% of pregnant women with preeclampsia had hypothyroidism, 71.42% had subclinical hypothyroidism and the rest had overt hypothyroidism. [12]. Another studies showed that %16.7 and %43.7 of preeclamptic women had subclinical and overt hypothyroidism, respectively [13]. Subclinical hypothyroidism is defined by an increase in TSH concentration and normal FT4 concentration, and overt hypothyroidism is defined by an increase of TSH level and decrease free thyroxine [5].

The mechanism of hypothyroidism in pre-eclamptic women was not well understood. However, according to various theories, this mechanism in preeclampsia may be related to a decrease in plasma protein concentration and an increase in endothelin levels [14]. In addition, the high circulation of estrogens can change thyroid function. Also, decreased thyroid function may be due to anti-angiogenic factors in preeclampsia that reduce nitric oxide production [15, 16]. This in turn decreases capillary flow of thyroid which could lead to hypothyroidism [8]. According to various studies, changes in thyroid function tests can affect the outcome of pregnancy [17, 18]. The results of a survey showed that subclinical hypothyroidism (SCH) is almost twice as likely to cause severe preeclampsia [5]. Early subclinical hypothyroidism in pregnancy is a risk factor for the premature rupture of membranes PROM [19]. Another study found that mothers with SCH had increased risks for spontaneous abortions [20], intrauterine growth restriction (IUGR) and low birth weight(LBW) [21].

During a normal pregnancy, changes in thyroid function are well documented, but information on thyroid function in complicated pregnancies is scant [22, 23]. The association of preeclampsia with thyroid function has been studied but with different results. However, Due to the high prevalence of preeclampsia and the contradictory findings of thyroid function tests in pre-eclampsia, the aim of this study was to Meta-analyze the data examining changes in thyroid function tests in preeclampsia. The results of the data combination may provide useful information for patient counseling and clinical management of women with preeclampsia.

### Objectives

The purpose of this systematic review and meta-analysis is to evaluate of thyroid function test abnormalities in preeclampsia.

## Materials and methods

### Protocol and registration

This study was reported based on the Preferred Reporting Items for Systematic Reviews and Meta-Analyses (PRISMA) checklist for systematic review and meta-analysis. The report was prepared in accordance with PRISMA 2020 guidelines [24], and the completed PRISMA 2020 checklist can be found in supplemental appendix 1.

The study protocol was registered in the PROSPERO (Registration number: CRD42020213560).

### Search strategy for the identification of studies

A comprehensive electronic search in the databases PubMed/Medline (NLM), SCOPUS, and Web of Science Core Collection was carried out in 31 August 2021. Google Scholar was also used to search for online research-related articles that may not be in the search databases to increase the comprehensiveness of the search. Medical Subject Headings (Mesh) and Embase Subject Headings (Emtree) were used to finding keywords. Keywords that were obtained from Emtree or Mesh were included in our search strategy without any changes. The search terms used were: “pregnancy toxemia”, “toxemia of pregnancy”, “preeclampsia”, “pre-eclampsia”, “preeclamptic women”, “pre-eclamptic women”, “TSH”, “thyroid stimulating hormone”, “thyrotropin”, “T3”, “triiodothyronine”, “T4”, “thyroxine”, “tetraiodothyronine”, “hypothyroidism”, “hypothyroidism” and “thyroid”. These keywords were combined with “AND” or “OR” browsers during the advanced search (see Appendix 2 for electronic database search strings). A snowball search was also used to find relevant articles. In this method, reference lists of review articles and related studies were manually searched to identify other potentially eligible studies.

All searches were restricted to English language. References were managed with EndNote 8.0 software.

### Study selection

Two independent reviewers (MF and SF) screened identified eligible studies based on their titles and abstracts. Then, all potentially relevant full texts were read to produce a final list of included studies for rejection recorded. The list of studies rejected at this stage and reasons for rejection can be found in supplemental appendix 3.

Any disagreements were resolved with discussion or through adjudication by a third review (SHT). The results of Cohen’s kappa indicated a perfect agreement between two authors (Kappa = 0.84).

Studies were incorporated if they were: original and peer-reviewed researches; one author conducted the initial screening analysis. After removing duplicates and scanning the titles and abstracts of articles, those meeting the inclusion criteria were reviewed.

### Study eligibility criteria

Studies that met all of the following criteria in this review:

### Included criteria

- Compared TSH, T4 and T3 of normal pregnant with preeclamptic women (Known to be normotensive before pregnancy; gestational age 20 week or more; singleton pregnancy; no previous history of thyroid dysfunction);
- English studies;
- Published or in-press articles until August 31, 2021.

### Excluded criteria

- Articles without appropriate case or control groups
- Animal studies
- Case reports
- Letter to the editor without quantitative data
- Qualitative studies
- Systematic reviews and meta-analyses
- Abstract without sufficient data or full-text for quality assessment.

For prevention misleading conclusions about excluding abstracts or full-texts without sufficient data, the first author sent five emails (every three days) to correspondence of studies. unfortunately, she did not receive any replies.

### Extracted information

- Research information (the first author, geographic location, year of publication, research design, sample size, finding);
- Characteristics of the participants (age, gestational age, number of participants, inclusion and exclusion criteria);
- Comparison of the details (number of groups, results of TSH, T4 and T3 tests);

### Data extraction

First outcome measurement in this meta-analysis were TSH, TT3, TT4, FT3 and FT4. Additionally, age, gestational age, birth weight, BMI, SBP, DBP, and parity were considered secondary outcomes measurement. Data were carefully and independently extracted from all eligible studies by two reviewers (MF and SF according to the inclusion criteria mentioned above using a Microsoft Excel spreadsheet). The extracted data included study characteristics (e.g., first author name, year of publication, country, sample size, patient age and gestational age. Disagreement was resolved by discussion or consulting with a third reviewer (SHT).

### Risk of bias (quality) assessment

In this study for evaluating the quality of studies, was used the Newcastle–Ottawa Scale (NOS) for case–control and clinical trial studies. The NOS is the most commonly tool for assessing the quality of observational cohort and cross-sectional studies [25], and each study is awarded scores for eight items: selection (maximum 4 scores for cohort and 5 scores for cross-sectional studies), comparability (maximum 2 scores), and Exposure (maximum 3 scores). Scores of the quality of articles was used as variable for subgroup analysis.

### Quality of evidence

The Grading of Recommendations Assessment, Development and Evaluation (GRADE) approach was recommended by the Cochrane collaboration and can be used for assessing the quality of study outcome levels. There are four levels of certainty of evidence: high, moderate, low, and very low [26]. For the GRADE approach, we used the software GRADEpro,

### Effect size

In this study, effect sizes were the difference between the mean of thyroid function indices (TSH, TT4, TT3, FT4 and FT3) divided by their standard errors related. The effect size was calculated for each study that had the inclusion criteria and was eventually used for combination in the meta-analysis.

### Strategy for data synthesis

The standardized mean difference (SMD) and 95% confidence interval (CI) were applied to compare TSH, TT3, TT4, FT3 and FT4 in two groups. For each variable, to test SMD = 0, we used this formula:

$$Z=\frac{{\overline{X} }_{Case}-{\overline{X} }_{Control}}{\sqrt{\frac{{S}_{Case}^{2}}{{n}_{case}}+\frac{{S}_{Control}^{2}}{{n}_{control}}}}$$

Prior to the meta-analysis of the studies, a sensitivity test was performed and studies that were not within the confidence interval were excluded from the meta-analysis. To evaluate the heterogeneity between the studies, Cochran's Q test (p < 0.10 indicated significance) with I2 (0–25%: low heterogeneity; 25–50%: moderate heterogeneity; > 50 high heterogeneity). I^2^ measure the inconsistency across study and in general when ${\mathrm{I}}^{2}$ > 50%, a random-effects model and if I^2^ < 50%, a fixed effect model is suitable for pooling the effect size. Therefore, a random effects model (the DerSimonian and Laird method) was used to estimate the pooled effect size (EF) and each target variable. The results of meta-analysis were reported as a form of a forest plot which containing SMD and 95% CI.

To evaluate the degree of heterogeneity between the mean studies, Q-Cochran test (p < 0.1 as heterogeneity) and I^2^ index (I^2^ > 50% indicating heterogeneity between studies) were used. If significant heterogeneity was observed between studies, meta-analysis was performed in layers of variables such as age, gestational age, birth weight, BMI (Body Mass Index), SBP (Systolic Blood Pressure), DBP (Diastolic Blood Pressure), and parity. When heterogeneity was observed in the layers of each of the mentioned variables, we related the reasons for possible heterogeneity to the mentioned variables. We also performed meta-regression for variables whose results in the subgroup test are significant.

Egger’s regression test was used to evaluate the publication bias between simultaneous studies. Hence, when the potential publication bias observed, the fill and trim method was performed. Also, all meta-analyzes were performed in STATA software version 14.

We concluded that there is a potential bias among studies. Then we use the “trim and fill” method to consider the missing potential studies on both sides of the funnel piece and then recalculate the effect size considering the inclusion of the missing potential studies [27].

## Results

**Fig. 1:**
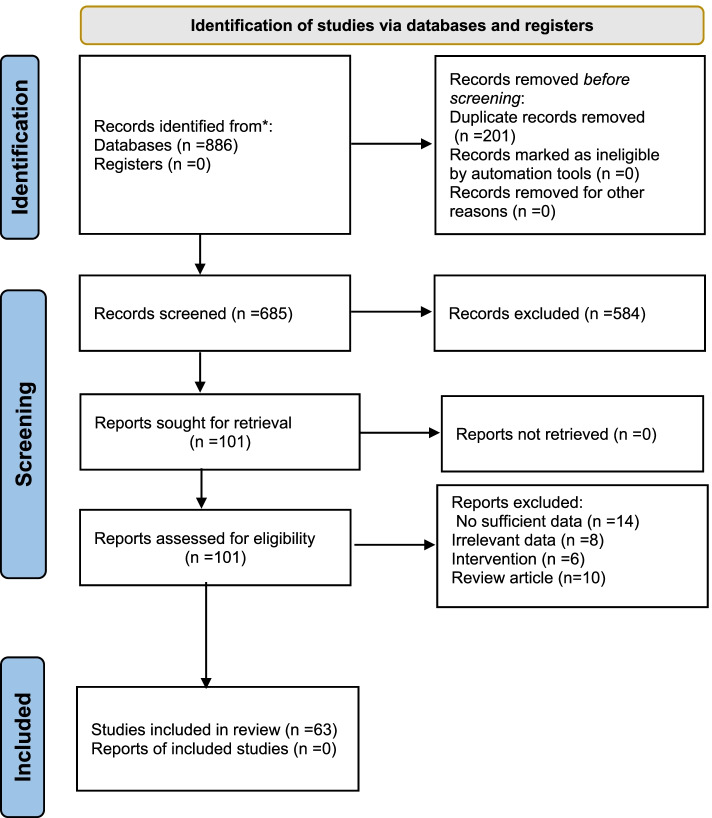
Flowchart of the selection of studies

Our search resulted in 886 articles. The article selection process was such that articles that did not qualify and did not meet the inclusion criteria were removed from the initial screening (based on the title or abstract of the article). Finally, we selected 63 articles for further reviewing (Fig. 1).

All of the studies selected after the search process met the inclusion criteria and had high quality according to Newcastle checklist. Mean age was 26.59 years in preeclamptic group and 25.67 in normal pregnant women. Mean gestational age was 35.32 years in preeclampsia and 38.2 in normal pregnancy.

The final study population comprised 21,528 pregnant women participants. Of whom 4765 (22.13%) of pregnant women were preeclamptic and 16,763 (77.87%) normotensives. Included study characteristics are summarized in Table 1.

**Table 1:** Characteristics of included studies in the meta-analysis

First author	Published Year	Country	Study design	Sample (Case/ Control)	Age (Case/ Control)	Gestational age (Case/ Control)	Mean TSH (Case/ Control)	Mean TT3 (Case/ Control)	Mean TT4 (Case/Control)	Mean FT3 (Case/ Control)	Mean FT4 (Case/ Control)	Quality Score
Muraleedharan [28]	2021	India	Case–control	40/40	24.98/24.55	34.50/ 34.50	3.76/2.30	1.28/1.62	11.59/13.63	2.12/2.43	1.16/1.33	8
Maduka [29]	2020	Nigeria	Case–control	40/40	31.5/29.5	N/A^a^	0.95/1.3	290/350	33.2/19.35	7.00/7.65	11.3/13.5	7
Abdal latief [30]	2020	Egypt	Case–control	30/ 30	23.69/ 25.30	34.14/ 34.07	5.18/1.96	N/A	N/A	4.07/3.53	0.99/1.12	8
Patil(a) [31]	2020	India	cross sectional	40/40	23.25/23.73	34.33/36.15	3.99/2.32	N/A	N/A	3.06/3.41	2.26/2.23	7
Patil(b) [31]	2020	India	cross sectional	40/40	22.25/23.73	33.10/36.15	5.66/2.32	N/A	N/A	2.77/3.41	2.34/2.23	7
Kumar [32]	2020	India	Case–control	100/100	22..02/ 22.11	34.54/ 36.13	4.21/ 1.98	118.11/131	7.84/ 11.87	N/A	N/A	8
Misra [33]	2020	India	Case–control	80/90	24.33/23.00	36.7/37.6	5.07/3.30	N/A	N/A	2.67/2.56	1.15/1.00	8
Bozkurt [34]	2020	Turkey	Retrospective	45/72	28.5/27.1	N/A^a^	2.4/2.3	N/A	N/A	2.6/2.6	0.9/ 0.9	7
Bozkurt [34]	2020	Turkey	Retrospective	27/72	30.3/27.1	N/A^a^	2.2/2.3	N/A	N/A	2.3/2.6	0.9/ 0.9	7
Marwa(a) [35]	2019	Iraq	Case–control	29/48	32.95/33.98	32.95/33.98	2.76/2.44	1.32/1.53	7.65/8.95	N/A	N/A	8
Marwa(b) [35]	2019	Iraq	Case–control	29/48	32.95/33.98	32.95/33.98	3.82/2.44	1.32/1.53	7.40/8.95	N/A	N/A	8
Prashanthi [36]	2019	India	Case–control	100/50	25.78/ 25.10	30.35/34.30	3.40/2.19	0.87/1.64	9.34/11.31	N/A	N/A	7
Tariq [37]	2018	Pakistan	Case—control	50/50	27.8/26.6	34.6/35.5	3.75/2.33	151.34/195.66	11.33/14.56	N/A	N/A	8
Sattar [3]	2018	Pakistan	Case–control	17/16	26.06/25.12	N/A	6.56/ 3.28	1.19/ 1.22	11.09/13.96	2.67/ 2.94	2.81/ 2.91	7
Murmu [38]	2018	India	Case–control	100/50	32.95/33.98	32.95/33.98	2.41/1.50	N/A	N/A	1.42/1.60	0.933/1.08	7
Chowdhary [39]	2018	India	Case–control	50/50	23.57/22.9	33.92/33.98	6.19/ 2.22	5.29/ 9.46	0.92/ 1.41	N/A	N/A	8
Ban(a) [40]	2018	Iraq	Case–control	50/100	25.1/25.0	38.8/39.1	3.23/2.1	2.30/2.70	1.46/1.79	N/A	N/A	8
Ban(b) [40]	2018	Iraq	Case–control	50/100	25.4/25.0	36.3/39.1	4.40/2.10	2.00/2.70	1.39/1.79	N/A	N/A	8
Amin [41]	2018	Pakistan	Case–control	40/40	N/A	N/A	2.65/2.25	N/A	N/A	1.44/1.48	8.75/10.09	7
Rani [42]	2018	India	Case–control	40/35	25.34/25.63	35.67/37.7	4.10/2.69	N/A	N/A	1.37/1.83	8.08/9.18	7
Murmu [43]	2018	India	Case–control	100/50	32.95/33.98	N/A	2.41/1.50	N/A	N/A	1.42/1.60	0.93/1.08	8
Muraleedharan(a) [16]	2017	India	Case–control	22/40	24.3/ 24.6	34.5/35.5	3.33/2.30	1.29/1.62	11.66/13.63	2.18/2.43	1.22/1.33	8
Muraleedharan(b) [16]	2017	India	Case–control	18/40	25.8/24.6	34.5/35.5	4.28/2.30	1.26/1.62	11.51/13.63	2.05/2.43	1.09/1.33	8
Grammatikakis [44]	2017	Greece	Retrospective analysis	60/60	29.2/28.8	37.1/38.6	4.40/2.40	N/A	N/A	3.45/2.80	0.70/0.70	7
Harshvardhan [14]	2017	India	Case–control	50/50	25.6/24.4	N/A	5.36/3.48	N/A	N/A	2.24/1.99	1.13/1.00	7
Dineshkumar [45]	2017	India	Case–control	40/40	27.4/26.64	N/A	2.79/2.42	N/A	N/A	1.54/ 1.60	10.89/11.10	7
Haldar [13]	2017	India	Case–control	100/100	N/A	N/A	4.39 /1.69	8.99/8.91	0.91/0.92	N/A	N/A	8
Jain [22]	2017	India	Case–control	40/40	26.15/26.19	N/A	8.64/4.65	11.31/14.36	150.62/ 195.6	N/A	N/A	8
Businge(a) [46]	2017	Congo	Case–control	200/150	32.4/33.5	26.9/37.7	4.10/2/70	1.30/1.20	10.70/9.90	N/A	N/A	7
Businge(b) [46]	2017	Congo	Case–control	200/150	32.4/33.5	32.5/37.7	6.60/2.70	1.50/1.20	11.40/9.90	N/A	N/A	7
Rajalaksmi [47]	2016	India	cross sectional	200/200	24.36/ 23.99	34.7/34.9	2.90/2.25	N/A	N/A	2.21/2.4	1.24/1.35	8
Reddy [48]	2016	India	Case–control	50/50	22.94 /22.17	35.85/ 33.54	5.22/3.37	1.40/1.36	11.99/10.28	N/A	N/A	8
Tadas [49]	2016	India	Case–control	50/50	27.8/26.6	34.6/35.5	3.75/ 2.33	11.33/14.56	151.34/195.66	N/A	N/A	8
Chauhan [50]	2016	India	Case–control	50/50	27.8/26.6	34.6/35.5	3.75/ 2.33	151.34/ 195.66	11.33/ 14.56	N/A	N/A	8
Chaudhary [39]	2016	India	Case–control	60/60	23.57/22.9	33.92/33.98	4.49/ 2.95	1.10/ 1.47	6.54/ 8.46	N/A	N/A	8
Procopciuc(a) [51]	2016	Romania	Case–control	57/131	29.07/28.41	34.72/38.79	3.26/2.34	N/A	N/A	2.44/2.85	1.19/0.97	7
Procopciuc(b) [51]	2016	Romania	Case–control	32/131			5.24/2.34	N/A	N/A	2.28/2.85	1.54/0.97	7
Umadevi [52]	2015	India	Case—control	200/200	24.36/ 23.99	34.7/ 34.9	2.90/ 2.25	N/A	N/A	2.21/ 2.4	1.24/ 1.35	8
Thanna [9]	2015	India	Case–control	30/30	25.25 /24.34	N/A	7.89 /3.22	1.18 /1.27	9.78/8.96	N/A	N/A	8
Sogani [15]	2015	India	Case–control	35/35	22.94/ 23.17	35.85/ 38.54	6.22/3.35	1.39/1.38	10.99/10.26	N/A	N/A	8
Rafeeinia [53]	2015	Iran	Case–control	50/50	26.5 /27.1	31.24 /30.17	2.30/1.82	4.00/3.90	5.03/5.02	N/A	N/A	8
Deshpande [54]	2015	India	Case–control	100/100	23.08/ 22.78	36.99/ 38.07	3.14/1.92	N/A	N/A	3.08/3.49	0.89/0.86	7
Elhaj(a) [55]	2015	Sudan	Case–control	55/55	28.1 /27.4	38.4/ 37.7	1.30/2.30	N/A	N/A	1.10/0.80	2.00/0.70	8
Elhaj(b) [55]	2015	Sudan	Case–control	55/55	29.1 /27.4	38.4/ 36.1	1.50/2.30	N/A	N/A	0.90/0.80	2.10/0.70	8
Sheela [56]	2015	India	Case–control	50/50	23.64/ 23.17	37.91/ 38.28	6.15/2.45	1.37/1.56	9.87/11.61	N/A	N/A	8
Satyanarayan [57]	2015	India	Case–control	30/30	N/A	N/A	7.22/2.48	1.25/1.21	10.16 /9.03	N/A	N/A	7
Camejo [58]	2014	Venezuela	Case–control	20/20	28.83/25.3	33.13 /35.3	2.60/2.50	N/A	N/A	2.99/3.35	1.00/1.00	8
Kaveti [59]	2014	India	Case–control	30/30	22.6/22.76	33.53/ 32.86	3.15/1.17	2.66/2.15	16.53/14.45	N/A	N/A	8
Naykı [23]	2014	Turkey	Case–control	50/30	26.84/ 28.3	35.44/ 38.03	2.41/2.51	N/A	N/A	3.23/2.96	1.58/1.28	7
Bayejid [60]	2014	Bangladesh	Case–control	27/25	25.04 /26.00	38.36/ 34.11	5.09/2.00	1.43/ 1.86	131.90/ 150.90	N/A	N/A	7
Manjunatha [61]	2014	India	Case–control	30/30	24/ 24	N/A	7.22/2.48	1.25/1.21	10.16/9.03	N/A	N/A	7
Kurlak [62]	2013	England	Cross sectional	23/27	32/29	36.4/39.9	3.4/2.5	N/A	N/A	3.7/3.7	11.2/11.9	8
Khanam [63]	2013	Bangladesh	Cross sectional	52/52	26.15/ 26.19	34.3/ 35.1	4.14/ 2.75	1.95/ 1.98	128.38/ 126.37	N/A	N/A	7
Das [64]	2013	India	Case–control	30/30	26 /27	36 /37	3.82/2.12	2.13/ 0.85	12.61/7.02	3.51/2.62	2.36/1.14	8
Monika [65]	2013	India	Case–control	25/25	N/A	N/A	3.59/2.93	1.83/2.18	101.46/101.47	N/A	N/A	8
Kharb(a) [18]	2013	India	Case–control	50/100	23.08	38.67	3.42/2.00	136.82/134.00	10.84/12.14	N/A	N/A	8
Kharb(b) [18]	2013	India	Case–control	50/100	23.01/ 23.04	36.87/ 39.09	5.63/2.00	119.64/134.00	9.39/12.14	N/A	N/A	8
Männistö [66]	2013	Finland	Prospective population-based cohort	381/4540	28.8/27.0	N/A	1.11/ 1.03	N/A	N/A	5.35/5.10	5.10/15.33	7
Procopciuc [67]	2012	Romania	Cohort	50/50	28.58 /28.04	35.36 /38.64	2.76/2.19	N/A	N/A	2.63/2.91	1.11/0.88	8
Alavi [68]	2012	Iran	Case–control	48/50	22.38/ 22.18	37.02/ 38.26	2*.*03/2*.*17	152*.*5/175*.*36	8*.*72/9*.*39	N/A	1*.*28/1.00	8
Riah(a) [69]	2012	Egypt	Prospective cross sectional	20/20	27.0/27.4	37.3/38.7	2.80/2.06	N/A	N/A-	1.40/1.50	0.90/1.00	8
Riah(b) [69]	2012	Egypt	Prospective cross sectional	20/20	28.9/27.4	36.5/38.7	4.30/2.06	N/A	N/A	1.50/1.50	1.01/1.00	8
Khadem [10]	2012	Iran	Case–control	40/40	28.0/26.0	35.57/38.5	3.51/3.10	N/A	N/A	3.51/1.41	0.95/0.96	8
Dhananjaya [70]	2011	India	Case–control	25/25	24.08/24.32	N/A	8.42/ 2.44	10.46/ 9.93	1.15/ 1.28	N/A	N/A	7
Al-Naqeeb(a) [71]	2010	Iraq	Case–control	37/30	30.05	N/A	1.14/1.12	0.89/1.21	10.81/8.69	N/A	N/A	7
Al-Naqeeb(b) [71]	2010	Iraq	Case–control	53/30	27.08/28.83	N/A	2.61/1.12	2.71/1.21	7.93/8.69	N/A	N/A	7
Obiero [72]	2010	Kenya	Case–control	35/35	28.7/28.1	32.1/31.4	2.51/1.53	N/A	N/A	1.94/2.04	12.39/14.01	7
Sardana [73]	2009	India	Case–control	100/100	23.7/23.04	37.77/39.09	4.52/2.00	128.23/134	10.12/12.14	N/A	N/A	8
Pasupathi [74]	2009	India	Case–control	30/30	27/28	35/36	5.25/ 3.89	1.85/ 2.17	12.75/ 12.62	2.72/ 3.57	2.42/ 2.38	7
Kumar [75]	2005	India	Case–control	82/82	28.4/27.5	34.3/35.1	4.6/ 2.5	N/A	N/A	3.1/ 2.7	0.8/ 0.9	8
Larijani(a) [76]	2004	Iran	Case–control	17/42	26.97/27.09	35.67/34.08	1.59/1.04	203.41/209.71	10.71/13.5	3.55/4.71	2.98/6.44	8
Larijani(b) [76]	2004	Iran	Case–control	22/42	27.07/27.09	34.02/34.08	1.54/1.04	203.13/209.71	12.73/13.5	5.45/4.71	8.82/6.44	8
Basbug [77]	1999	Turkey	Case–control	37/20	23.7/24.8	35.4/38.3	2.96/1.55	141.16/180.58	10.00/13.76	2.41/3.32	1.1/1.45	7
Khaliq [78]	1999	India	Case–control	32/10	N/A	N/A	3.77/ 2.34	150.62/ 195.6	11.31/ 14.36	N/A	N/A	7
Kaya [79]	1994	Turkey	Case–control	45/45	27.6/27.0	35.6/ 37.2	2.00/ 1.50	156/ 196	11.8/ 14.7	N/A	N/A	8
Lao [80]	1990	Hong Kong	Case–control	39/24	N/A	N/A	3.9/2.5	1.9/2.1	118.8/131.6	2.8/3.3	12.5/16.3	7
Lao [81]	1988	Hong Kong	Case–control	24/24	28.4/27.5	37.5/39.3	3.9/2.5	1.9/2.1	118.8/131.6	2.8/3.3	12.5/16.3	8

(a) Case group: Mild preeclampsia, (b) Case group: Severe preeclampsia

^a^N/A: Not/Available

### Sensitivity analysis

Initially, we performed sensitivity analyses by consecutive removal of a study at a time to evaluate the change in the pooled SMD and 95% CI of each thyroid hormone. The results of sensitivity analysis showed that the pooled effect size of thyroid hormones is 95% confidence interval. In other words, when each study was removed, there was no significant change in the pooled SMD.

**Fig. 2:**
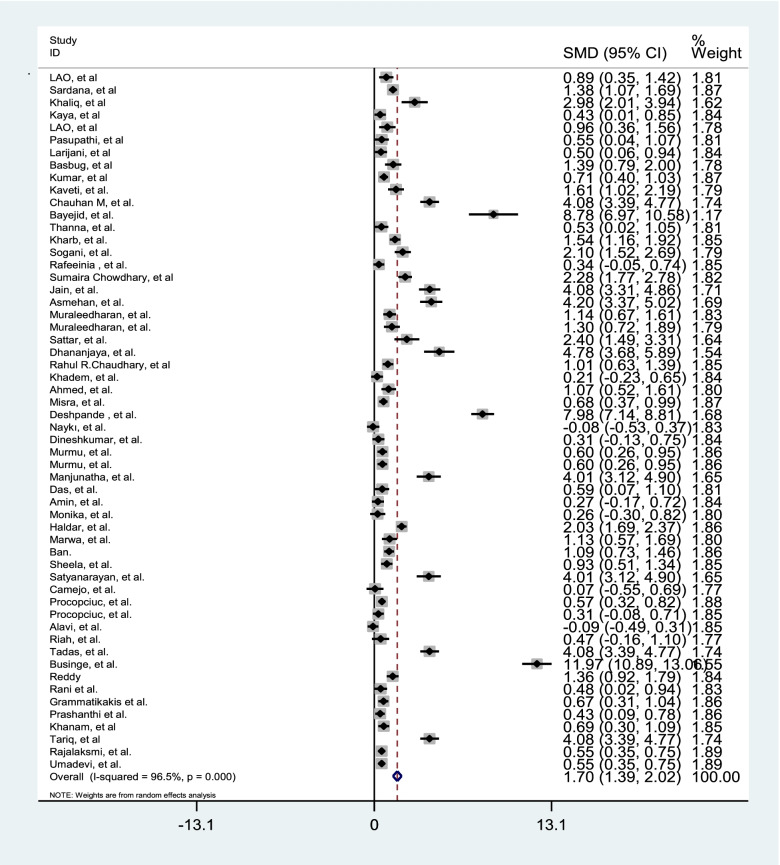
Forest plots showing standard mean differences (SMD, 95% CI) for TSH

**Fig. 3:**
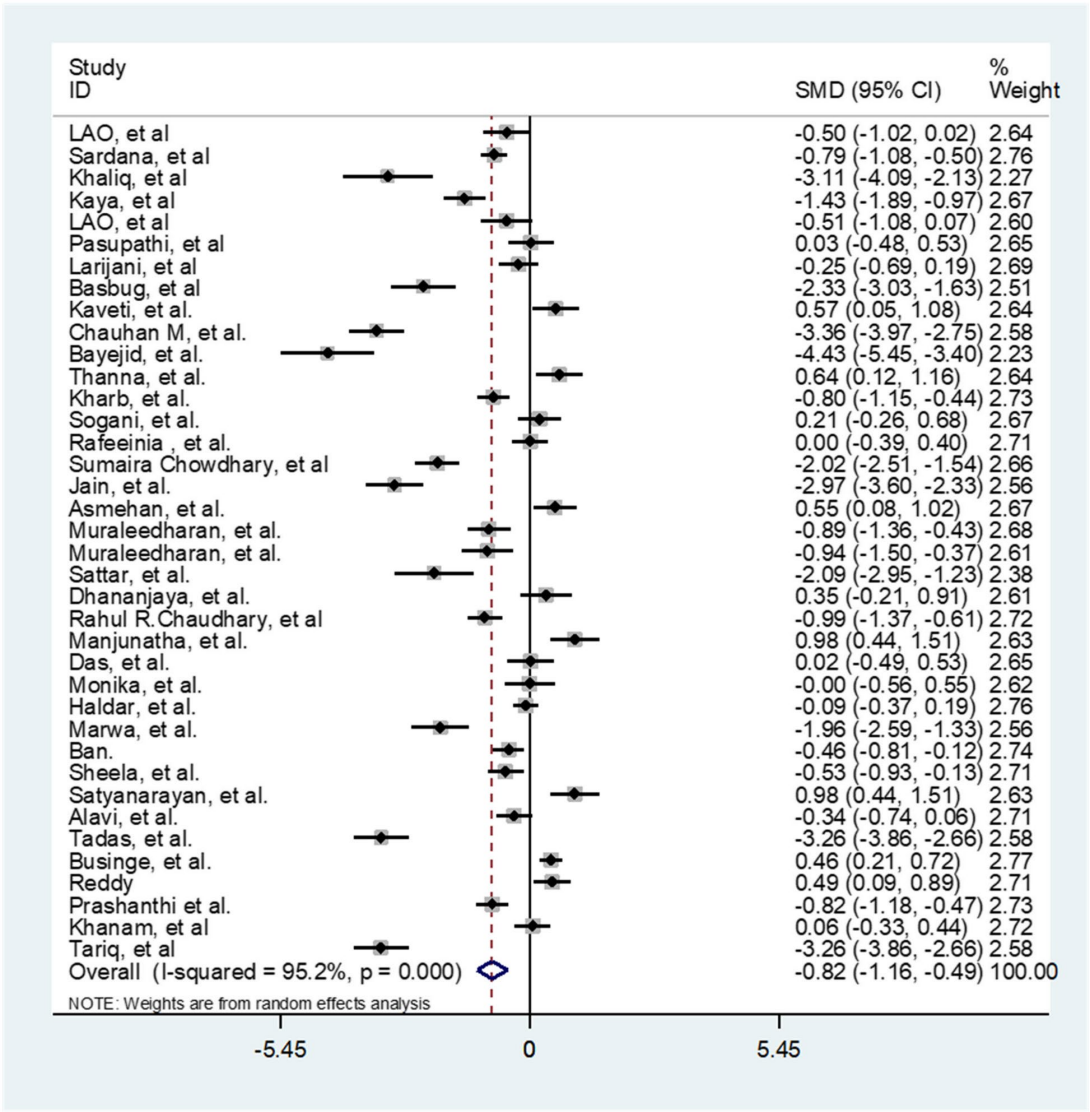
Forest plots showing standard mean differences (SMD, 95% CI) for TT4

**Fig. 4:**
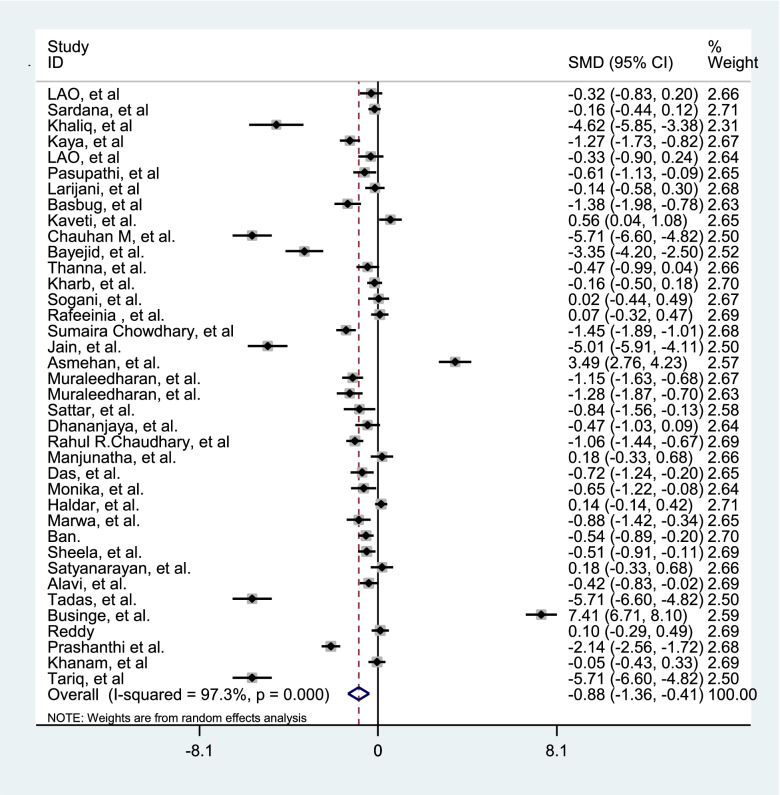
Forest plots showing standard mean differences (SMD, 95% CI) for TT3

Since there was a significant heterogeneity between studies, subgroup analysis was performed. The subgroup analysis was performed according to the age, gestational age, birth weight, BMI, SBP, DBP, parity (Table 2). In order to evaluate the impact of heterogeneous studies on the pooled estimates, we conducted a sensitivity analysis. For this purpose, we excluded studies serially and obtained pooled estimates from the remaining studies. This enabled us to evaluate whether single studies with highly heterogeneous results were affecting the overall pooled estimates. Results of this study, there is a potential bias among the studies. We then use the "draw and fill" method to consider the missing potential studies on either side of the funnel piece and recalculate the effect size by considering the inclusion of the lost potential studies For meta-analysis of compare gestational age of preeclampsia and normal pregnant women 37 studies were applied. The results showed in Table 3. Fifty-six studies comparing TSH were performed in preeclampsia and normal pregnant women. The results presented in Table 3 and Fig. 2. Thirty-eight studies have been performed to estimate the total SMD of TT4 and TT3. We found TT4 (Table 3 and Fig. 3) and TT3 (Table 3 and Fig. 4) for preeclampsia women significantly were less than normal women. Twenty-two studies were performed to compare FT4 in preeclampsia and normal pregnant women. The results showed that there was no statistically significant difference between the two groups (Table 3).

**Table 2:** The effect of some covariates on factors of thyroid hormones in preeclamptic and normal pregnant women

Factors	Covariates	Preeclamptic women			Normal pregnant women		
		β	*t*	*P*	β	*t*	*P*
TSH	Age	-0.11	-1.36	.180	-0.19	-0.47	.642
	Gestational Age	-0.43	-0.68	.534	-0.006	-.09	.927
	Birth weight	-0.001	-0.39	.702	0.001	-0.76	.463
	BMI	-0.19	-1.45	.192	0.04	0.37	.722
	SBP	-0.07	-1.52	.149	-0.002	-0.12	.903
	DBP	-0.02	-0.41	.690	0.98	1.73	.105
	parity	-0.24	-0.75	.457	0.34	0.77	.459
	Quality	-0.33	-0.56	0.58	-0.22	-0.97	.34
TT3	Age	1.08	0.23	.817	1.22	0.42	.679
	Gestational Age	3.77	0.92	.370	2.71	0.43	.699
	Birth weight	0.006	0.52	.630	-0.004	-0.36	.735
	BMI	-.48	-1.51	.207	-0.46	-0.95	.395
	SBP	-0.50	-0.24	.812	0.13	0.22	.834
	DBP	-1.50	-1.0	.339	3.18	0.75	.469
	parity	-20.0	-4.19	.052	-10.2	-0.98	.431
	Quality	9.90	0.46	.650	9.21	0.34	.74
TT4	Age	-0.67	-0.34	.736	0.33	0.29	.777
	Gestational Age	-0.21	-0.10	.921	2.23	0.79	.438
	Birth weight	-0.01	-0.11	.917	0.002	0.02	.987
	BMI	0.48	0.70	.524	0.68	0.82	.640
	SBP	-0.34	-0.65	.532	0.02	0.08	.935
	DBP	-0.06	-0.12	.903	-2.62	-1.48	.170
	parity	-10.1	-1.80	.214	-9.05	-2.88	.102
	Quality	3.55	1.31	0.198	3.37	0.28	.780
FT4	Age	-0.05	-0.43	.669	-0.0001	0.001	.998
	Gestational age	-0.06	-1.64	.121	-0.07	-1.82	.090
	Birth weight	-0.001	-0.82	.240	-0.002	-0.22	.836
	BMI	0.40	1.17	.363	1.76	1.32	.269
	SBP	0.46	2.81	.**031**	0.09	0.36	.735
	DBP	0.16	0.61	.570	0.44	1.14	.305
	parity	0.08	0.79	.472	0.15	1.48	.199
	Quality	0.15	0.58	.57	1.85	1.15	.26
FT3	Age	-0.16	-2.51	***.022***	-0.14	-2.26	***.037***
	Gestational age	0.07	0.45	.660	-0.12	-0.79	.442
	Birth weight	-0.001	-0.19	.851	0.001	0.82	.459
	BMI	-0.24	-0.74	.539	-0.30	-0.80	.482
	SBP	-0.09	-2.31	.***043***	-0.01	-0.22	.836
	DBP	0.03	0.49	.642	0.04	0.44	.680
	Parity	0.07	0.47	.659	0.32	0.35	.744
	Quality	-0.15	1.88	0.07	-0.17	-0.49	.63

**Table 3:** Comparison of gestational age and the thyroid hormones in preeclamptic and normal pregnant women

Characteristics	n	Preeclamptic (Mean)	Normal (Mean)	SMD (95% CI)	Test of SMD = 0		Heterogeneity		Egger’s test	
					**z**	**p**	${{\varvec{I}}}^{2}$**(%)**	**p**	**t**	**P**
**Gestational age**	37	35.32	38.2	-0.50(-0.99,-0.46)	5.33	** < .001**	94.0	< .001	2.11	.170
**TSH**	56	3.55	1.85	1.70(1.39,2.02)	10.62	** < .001**	96.5	< .001	6.09	< 0.001
**TT4**	38	25.26	39.12	-0.82(-1.16,-0.49)	4.80	** < .001**	95.2	< .001	-3.32	.002
**TT3**	38	63.55	62.22	-0.88(-1.36,-0.41)	3.63	** < .001**	97.3	< .001	-1.89	.113
**FT4**	22	3.83	4.99	0.002(-0.27, 0.27)	0.02	.980	90.8	< .001	-1.50	.160
**FT3**	28	2.88	0.90	-0.59(-0.91,-0.27)	3.19	** < .001**	94.4	< .001	-2.46	.043

**Fig. 5:**
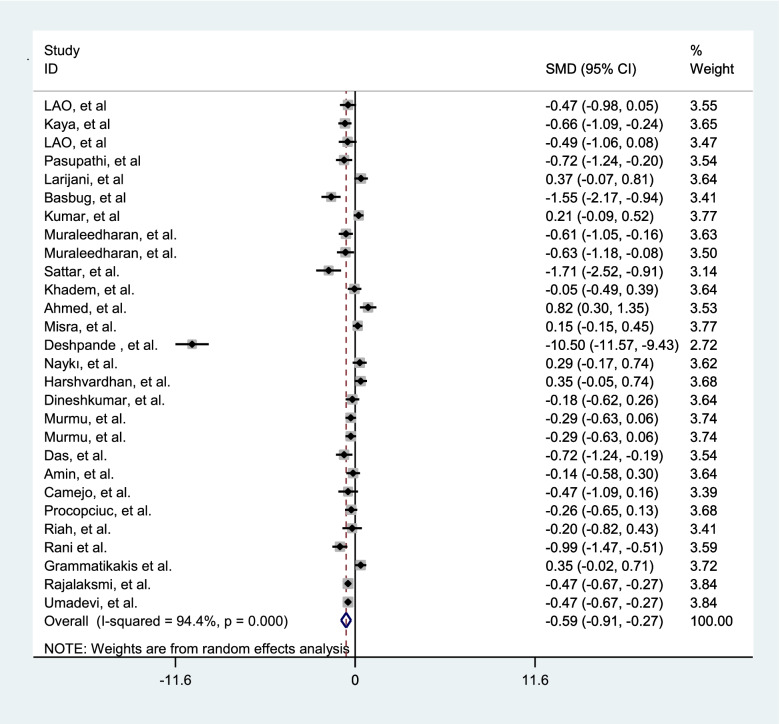
Forest plots showing standard mean differences (SMD, 95% CI) for FT3

Based on the results of pooled SMD in 28 studies, FT3 was significantly lower in women with preeclampsia than in normal women (Table 3 and Fig. 5).

**Fig. 6:**
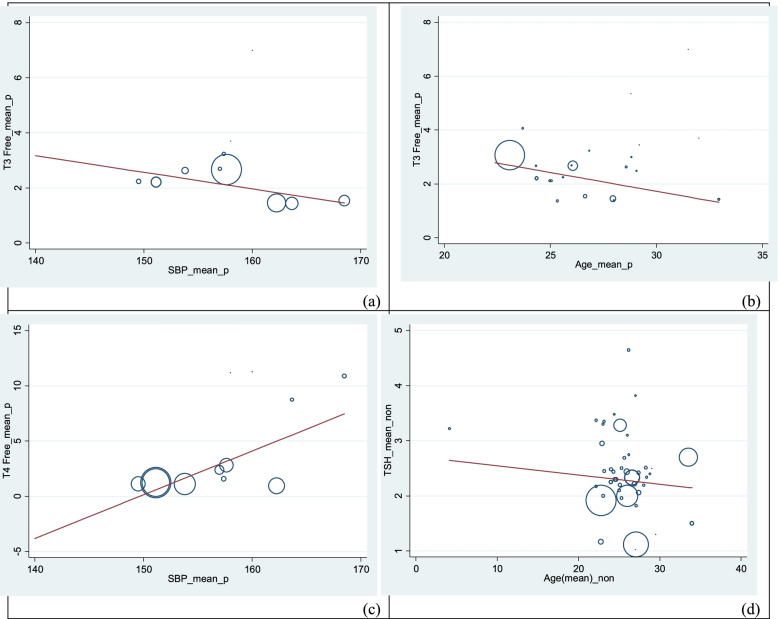
Meta-regression of SBP vs T3free (**a**), age vs T3 free (**b**), SBP vs T4 free (**c**) and age vs T3 free (**d**). (vs., versus)

For preeclamptic women, meta-regression results showed that with increasing age (β = -0.16, *p* = 0.022) and SBP (β = -0.09, *p* = 0.043) significantly FT3 was decreased. Also, FT4 increased significantly with increasing SBP (β = 0.46, *p* = 0.031). In the group of normal pregnant women, FT3 decreased significantly with age (β = -0.14, *p* = 0.037) (Table 2 and Fig. 6).

### Publication bias

Publication bias in meta-analysis was evaluated by performing Egger rank correlation test for statistical analysis of plots symmetry. The results showed that except for TSH (t = -9.09, p < 0.001), TT4 (t = -3.32, *p* = 0.002) and FT3 (t = -2.09, *p* = 0.043) no observed publication bias among studies for other variables.

### Quality of Evidence

We are moderately confident in the effect estimate TT4, FT4 and FT3: the true effect is likely to be close to the estimate of the effect, but there is a possibility that it is substantially different For TT3, our confidence in the effect estimate is limited: The true effect may be substantially different from the estimate of the effect. Also, for TSH, we have very little confidence in the effect estimate: the true effect is likely to be substantially different from the estimate of effect.

According to results of GRADE assessment the risk of bias, inconsistency, indirectness and imprecision for all effect sizes were not serious. The detailed table is presented in Table 4.

**Table 4:** Overall Quality of Evidence for Thyroid function tests in preeclampsia women with healthy pregnant women

**Outcome № of participants (studies)**	**Risk of bias**	**Inconsistency**	**Indirectness**	**Imprecision**	**Publication bias**^a^	**Overall certainty of evidence**	**Relative effect** **(95% CI)**	**What happens**
**Thyroid Stimulating Hormone (TSH**) № of participants: 4765 cases 16,763 controls 4765/ exposed 16,763/ unexposed (56 observational studies)	Not serious	Not serious	Not serious	Not serious	Publication bias strongly suspected	⨁◯◯◯ Very low	**1.7** (1.39 to 2.02)	The evidence is very uncertain about the effect of preeclampsia on thyroid
**Total Thyroxine (TT4)** № of participants: 4765 cases 16,763 controls / exposed / unexposed (38 observational studies)	Not serious	Not serious	Not serious	Not serious	None	⨁⨁⨁◯ Moderate	**-0.82** (-1.16 to -0.49)	Preeclamptic women may result in a large reduction in total Thyroxine
**Total Triiodothyronine (TT3)** № of participants: 4765 cases 16,763 controls / exposed / unexposed (38 observational studies)	Not serious	Not serious	Not serious	Not serious	None	⨁⨁◯◯ Low	-0.**88** (-1.36 to -0.41)	The evidence suggests preeclamptic women reduces free T4 slightly
**Free T4 (FT4)** № of participants: 4765 cases 16,763 controls / exposed / unexposed (22 observational studies)	Not serious	Not serious	Not serious	Not serious	None	⨁⨁⨁◯ Moderate	**0.002** (-0.27 to 0.27)	The evidence suggests preeclamptic women reduces free T4 slightly
**Free T3 (FT3**) № of participants: 4765 cases 16,763 controls / exposed / unexposed (28 observational studies)	Not serious	Not serious	Not serious	Not serious	None	⨁⨁⨁◯ Moderate	**-0.59** (-0.91 to -0.27	The evidence suggests preeclamptic women reduces free T3 slightly

^a^Publication bias was assessed in the meta-analysis by performing Egger’s rank correlation test to examine the symmetries of the plots statistically. The results showed that except for TSH (t =—9.0 9, *p* < 0.001)

## Discussion

In this meta-analysis study, for the first time, thyroid function tests in preeclampsia were compared with normal pregnant women. The results of this study showed that the mean TSH level is higher in preeclamptic women, but TT4, TT3 and FT3 are lower. Additionally, there was no statistically significant difference in FT4 between the two groups.

The results of this study showed that the higher TSH mean level in preeclamptic patients, which is consistent with other studies [35, 64, 82]. Even though placental function abnormalities can interfere with estrogen production, leading to decrease of TBG levels and it can be a reason for elevated TSH secretion by anterior pituitary [59, 61] but in the some studies, TSH level in preeclamptic women was not higher than normal pregnant women [3, 41].

Consistent with our findings, several studies have observed lower TT4 and TT3 levels in preeclampsia [7, 16, 35]. According to other studies, women with preeclampsia have increased antiangiogenic factors, which in turn reduce nitric oxide production. This in turn reduces thyroid capillary flow, which can lead to hypothyroidism [82]. In a normal pregnancy, estrogen increases serum TBG, but in preeclampsia, lack of estrogen production leads to a decrease in TBG. Also, placental dysfunction and decreased estrogen production in preeclampsia can reduce TT3 and TT4. [38, 73]. Also, in women with hypertension, the presence of protein-binding hormones in the urine and a decrease in serum albumin due to proteinuria can lead to a decrease in TT3 levels. Increased TSH levels in preeclampsia are associated with concomitant decreases in TT4, TT3 and endothelin levels. Accordingly, endothelial dysfunction is seen in extensive vasospasm and poor perfusion of many tissues such as the thyroid in preeclampsia. [73]. Other investigations have reported higher TT4 and TT3levels in preeclamptic women [9, 48].

Our finding observed FT3 in preeclamptic women was significantly different from normal pregnant. Low FT3 levels were observed in preeclamptic patients in a few studies, too [3, 33, 41, 58]. The hypothesis appropriate to these conditions is that the conversion of T4 to T3 is impaired in the liver, which could be the reason for the low level of FT3 in women with preeclampsia [30]. No difference in FT3 between the two groups was observed in other studies [30, 64, 82]. The findings of present study showed lower mean FT4 level in preeclamptic patients is similar to the results of other studies [30, 38, 41] while finding of some investigations showed a higher mean FT4 level in preeclampsia [14, 23].

In our study, results of meta-regression presented that in preeclamptic women with increasing SBP significantly FT3 was decreased. Also, FT4 increased significantly with increasing SBP. Maduka and et al. did not reported any correlation between FT3 or FT4 with systolic blood pressure in the preeclamptic women [29].

In another study, TSH is correlated positively with systolic blood pressure in preeclampsia [31] but this relationship did not find in this metanalysis. The differences observed in researches could be as a result of differences in the race, diets, and geographical locations of participants [29].In general, the results of current meta-analysis showed that an increase serum TSH with a meaningless difference in FT4 in preeclamptic women is called subclinical hypothyroidism [5]; this finding is supported by previous studies [42, 44, 64].

The relation between changes in thyroid function and preeclampsia may be reciprocal [15]. This means that the thyroid disorder is one of the predisposing causes for pre-eclampsia [57] and hypothyroidism is one of the pathophysiologic causes of pre-eclampsia.. Hypothyroidism can play an important role in smooth muscle contraction in the renal and systemic arteries, leading to increased peripheral vascular resistance, diastolic blood pressure, and decreased tissue perfusion [57, 60]. Therefore, the identification of thyroid abnormalities and their proper management can affect the incidence of preeclampsia [60]. It was suggested that evaluation of thyroid function could be useful in predicting preeclampsia [55]. In this regard, TSH plays a central role in screening and diagnosis of many thyroid disorders [5].

On the other hands, preeclampsia can be a key cause in the pathogenesis of hypothyroidism. Effects of preeclampsia in thyroid function is not yet elucidated, but increased levels of endothelin as a vasoconstrictor produced by vascular endothelium are involved in the pathogenesis of subclinical hypothyroidism in preeclampsia [44]. Out of 95 preeclamptic women, 44.2% were thyroid dysfunction with 38.9% patients had subclinical hypothyroidism and 4.2% overt hypothyroidism and 1% hyperthyroidism [83].

Nowadays, some researchers have cited the relationship between maternal and fetal complications with thyroid dysfunction is a problem of concern [5, 23, 33]. Therefore, obstetricians and endocrinologists should be increasingly aware of the potential complications of hypothyroidism in pregnancy [49] and should recommend thyroid function screening in pregnancy for early diagnosis; follow-up in the third trimester of pregnancy, especially in preeclamptic women and treatment of thyroid dysfunction to prevent further complications [50, 57, 61]. Fortunately, the evaluation of thyroid function tests (serum T3, T4 and TSH) is reliable, simple, economical and sensitive [65].

The results of this study showed that except for TSH, there was no diffusion bias between studies for other variables. These results could mean that only research that has published positive results and published negative or insignificant statistical studies will not be published because they see it as a failed researcher that is not true. [84].

The quality of evidence for TSH was rated as very low by the GRADE that any effect of preeclampsia on TSH is very uncertain. The quality of evidence for TT4, FT4, and FT3 were rated as moderate. It shows in future studies, preeclampsia has likely an important effect on them and may change the estimate. For TT3, the quality of evidence was rated as low which present future studies, preeclampsia is very likely to have an important effect on TT3 and is likely to change the estimate.

Researches showed drug therapy may reduce the complications of pregnancy in women with thyroid dysfunction and recommended thyroid function be checked in preeclamptic women [85, 86]. Further studies are needed to investigate the odds of preeclampsia among hypothyroid women.

Present study has several strengths. This is the first systematic review and meta-analysis about the relationship between preeclampsia and the thyroid function tests. Accordingly, the finding of current study showed more reliable and evident results than the separate studies. Moreover, included studies were performed in four continents of Asia, Africa, America and Europe. Forth, the results of this meta-analysis have important implications for care from preeclamptic women and screening of thyroid hormone levels during pregnancy. We used the GRADE approach to appraise the quality of evidence and assessed the risk of bias of studies.

The limitation of the current study is that the great majority of studies comprising the meta-analysis reported findings for mixed groups of mild and severe preeclampsia. However, few studies reported results separately for mild and severe. Accordingly, we could not find the relation between changes in thyroid function tests with the severity of preeclampsia.

## Conclusion

Findings of this meta-analysis indicated preeclamptic women were more at risk of changes in thyroid function tests. Therefore, thyroid function tests should be considered in preeclamptic women. Identification of changes in thyroid hormones in preeclampsia might be of help in preventing the thyroid disorders.

These results provide insights into the optimizing clinical decision-making strategies that should provide thyroid screening in women with preeclampsia.

## Supplementary Information

**Additional file 1: Supplemental appendix 1.** PRISMA 2020 Checklist.

**Additional file 2: Supplemental appendix 2.** Electronic database search strings.

**Additional file 3: Supplemental appendix 3**. Studies rejected at full-text review stage.

## Data Availability

All data analyzed during this study are included in this published article.

## Abbreviations

ACOG: American College of Obstetricians and Gynecologists
ATA: American Thyroid Association
BMI: Body Mass Index
CI: Confidence Interval
DBP: Diastolic Blood Pressure
EF: Effect size
FT3: Free Triiodothyronine
FT4: Free Thyroxine
GRADE: Grading of Recommendations Assessment, Development and Evaluation
HCG: Human Chorionic Gonadotropin
IUGR: Intrauterine Growth Restriction
LBW: Low birth weight
LT4: Levothyroxine
NOS: Newcastle–Ottawa Scale
PRISMA: Preferred Reporting Items for Systematic Reviews and Meta-Analyses
PROM: Premature Rupture Of Membranes
SBP: Systolic Blood Pressure
SMD: Standardized Mean Difference
SCH: Subclinical Hypothyroidism
TBG: Thyroxine Binding Globulin
TSH: Thyroid Stimulating Hormone
TT3: Total Triiodothyronine
TT4: Total Thyroxine
