# Supplementary material for: Association of thyroid function test abnormalities with preeclampsia: a systematic review and meta-analysis
Source: BMC Endocr Disord. 2022 Sep 26;22:240. doi: 10.1186/s12902-022-01154-9 (PMC9511725; doi:10.1186/s12902-022-01154-9)
Supplement: Supplementary file 2 — Additional file 2: Supplemental appendix 2. Electronic database search strings. [file 12902_2022_1154_MOESM2_ESM.docx]

**Supplemental appendix 2: Electronic database search strings**

| **PubMed/MEDLINE** | #1. Preeclampsia[mh] OR Toxemia[mh] OR Preeclampsia[tiab] OR Toxemia[tiab] OR Pre-Eclampsia[tiab] OR “Pregnancy Toxemias”[tiab] OR “Pregnancy Toxemia”[tiab] OR “Toxemia Of Pregnancy”[tiab] OR “Toxemia Of Pregnancies”[tiab] OR preeclamptic[tiab] OR Pre-eclamptic[tiab]  #2. (thyroid[mh] OR “thyroid gland”[mh] OR “thyroid Hormones”[mh] OR “thyroid Disease”[mh] OR TSH[tiab] OR “thyroid stimulating hormone”[tiab] OR thyrotropin[tiab] OR T3[tiab] OR triiodothyronine[tiab] OR T4[tiab] OR thyroxine[tiab] OR tetraiodothyronine[tiab] OR hyperthyroidism[tiab] OR hypothyroidism[tiab] OR thyroid[tiab] OR “thyroid gland”[ tiab] OR “thyroid Hormones”[ tiab] OR “thyroid Disease”[ tiab])  #3. (#1 AND #2) |
| --- | --- |
| **Web of science** | #1. Ti= (Preeclampsia OR Toxemia OR Pre-Eclampsia OR “Pregnancy Toxemias” OR “Pregnancy Toxemia” OR “Toxemia Of Pregnancy” OR preeclamptic OR Pre-eclamptic)  #2. Ti=(TSH OR “thyroid stimulating hormone” OR thyrotropin OR T3 OR triiodothyronine OR T4 OR thyroxine OR tetraiodothyronine OR hyperthyroidism OR hypothyroidism OR thyroid)  #3. #1 OR #2 |
| **Scopus** | ( TITLE-ABS-KEY ( tsh ) OR TITLE-ABS-KEY ( "thyroid stimulating hormone" ) OR TITLE-ABS-KEY ( thyrotropin ) OR TITLE-ABS-KEY ( t3 ) OR TITLE-ABS-KEY ( triiodothyronine ) OR TITLE-ABS-KEY ( t4 ) OR TITLE-ABS-KEY ( thyroxine ) OR TITLE-ABS-KEY ( tetraiodothyronine ) OR TITLE-ABS-KEY ( hyperthyroidism ) OR TITLE-ABS-KEY (hypothyroidism ) OR TITLE-ABS-KEY (thyroid ) AND TITLE-ABS-KEY ( preeclampsia ) OR TITLE-ABS-KEY ( toxemia ) OR TITLE-ABS-KEY ( pre-eclampsia ) OR TITLE-ABS-KEY ( “pregnancy AND toxemias” ) OR TITLE-ABS-KEY ( “pregnancy AND toxemia” ) OR TITLE-ABS-KEY ( “Toxemia of Pregnancy” ) OR TITLE-ABS-KEY ( “toxemia AND of AND pregnancies” ) OR TITLE-ABS-KEY ( preeclamptic ) OR TITLE-ABS-KEY ( pre-eclamptic ) ) |
