# Supplementary material for: Association of thyroid function test abnormalities with preeclampsia: a systematic review and meta-analysis
Source: BMC Endocr Disord. 2022 Sep 26;22:240. doi: 10.1186/s12902-022-01154-9 (PMC9511725; doi:10.1186/s12902-022-01154-9)
Supplement: Supplementary file 3 — Additional file 3: Supplemental appendix 3. Studies rejected at full-text review stage. [file 12902_2022_1154_MOESM3_ESM.docx]

**Supplemental Appendix 3: Studies rejected at full-text review stage**

| **No.** | **Study reference** | **Reason for rejection** |
| --- | --- | --- |
| **1** | Correlation between the TSHRc-Asp727Glu polymorphism and plasma thyroid stimulating hormone levels in Romanian preeclamptic women | No sufficient data |
| **2** | Evaluation of thyroid stimulating hormone and free Thyroxine and their correlations with other biomarkers in Sudanese pre-eclamptic cases | No sufficient data |
| **3** | Associations between Maternal Thyroid Function in Pregnancy and Obstetric and Perinatal Outcomes | No sufficient data |
| **4** | Subclinical hypothyroidism with preeclampsia. | No sufficient data |
| **5** | Preeclampsia, Soluble Fms-like Tyrosine Kinase 1, and the Risk of Reduced Thyroid Function: Nested Case-Control and Population-based Study | No sufficient data |
| **6** | The risk of pre-eclampsia according to high thyroid function in pregnancy differs by hCG concentration | No sufficient data |
| **7** | Comparison of thyroid hormone levels between normal and preeclamptic pregnancies | No sufficient data |
| **8** | History of preeclampsia is not associated with an increased risk of thyroid dysfunction | No sufficient data |
| **9** | Subclinical hypothyroidism with preeclampsia | No sufficient data |
| **10** | The risk of pre-eclampsia according to high thyroid function in pregnancy differs by hCG concentration | No sufficient data |
| **11** | Thyroid dysfunction in preeclampsia and related fetomaternal outcomes | No sufficient data |
| **12** | Severe pre-eclampsia and maternal thyroid function | No sufficient data |
| **13** | Comparison of serum levels of thyroid-stimulating hormone in preeclampsia and non-preeclampsia pregnant women referring to Karaj Kamali Hospital in 2018 | No sufficient data |
| **14** | The relationship between thyroid autoantibody positivity and abnormal pregnancy outcomes and miscarriage in euthyroid patients | No sufficient data |
| **15** | Hypothyroidism presenting as hypothermia following pre-eclampsia at 23 weeks gestation. Case report and review of the literature | Review |
| **16** | Autoimmune thyroid disease during pregnancy | Review |
| **17** | The impact of thyroid autoimmunity on IVF/ICSI outcome: a systematic review and meta-analysis | Review |
| **18** | Female Infertility and Serum Auto-antibodies: a Systematic Review | Review |
| **19** | Significance of (sub)clinical thyroid dysfunction and thyroid autoimmunity before conception and in early pregnancy: a systematic review | Review |
| **20** | Thyroid physiology and common diseases in pregnancy: review of literature. | Review |
| **21** | Thyroxine replacement for subfertile women with euthyroid autoimmune thyroid disease or subclinical hypothyroidism | Review |
| **22** | Thyroid antibodies and risk of preterm delivery: a meta-analysis of prospective cohort studies | Review |
| **23** | Effects of Levothyroxine on Pregnancy Outcomes in Women With Thyroid Dysfunction: A Meta-analysis of Randomized Controlled Trials | Review |
| **24** | Significance of (sub)clinical thyroid dysfunction and thyroid autoimmunity before conception and in early pregnancy: a systematic review | Review |
| **25** | The Impact of Subclinical Hypothyroidism on Adverse Perinatal Outcomes and the Role of Thyroid Screening in Pregnancy | Irrelevant data |
| **26** | The Joint Role of Iodine Status and Thyroid Function on Risk for Preeclampsia in Finnish Women: a Population-Based Nested Case-Control Study | Irrelevant data |
| **27** | Moor. maternal thyroid function at 11 to 13 weeks of gestation and subsequent development of preeclampsia. | Irrelevant data |
| **28** | Maternal thyroid function at 11 to 13 weeks of gestation and subsequent development of preeclampsia | Irrelevant data |
| **29** | Thyroid autoimmunity in pregnancy and its influences on maternal and fetal outcome in Iran | Irrelevant data |
| **30** | Maternal Early-Pregnancy Thyroid Function Is Associated With Subsequent Hypertensive Disorders of Pregnancy: The Generation R Study | Irrelevant data |
| **31** | Effect of thyroid dysfunction and autoimmunity on pregnancy outcomes in low risk population | Irrelevant data |
| **32** | Association between thyroid dysfunction and perinatal outcomes in women with gestational hypertension: a retrospective study | Irrelevant data |
| **33** | Pre-eclampsia, soluble fms-like tyrosine kinase 1, and the risk of reduced thyroid function: nested case-control and population based study | Interventional |
| **34** | Initiation timing effect of levothyroxine treatment on subclinical hypothyroidism in pregnancy | Interventional |
| **34** | Hypothyroidism and the increased risk of preeclampsia – interpretative factors? | Interventional |
| **36** | Effect of levothyroxine supplementation on pregnancy outcomes in women with subclinical hypothyroidism and thyroid autoimmunity undergoing in vitro fertilization/intracytoplasmic sperm injection: an updated meta-analysis of randomized controlled trials | Interventional |
| **37** | Hypothyroidism and the increased risk of preeclampsia – interpretative factors? | Interventional |
| **38** | Optimal management of hypothyroidism, hypothyroxinaemia and euthyroid TPO antibody positivity preconception and in pregnancy | Interventional |
